# Retinal tissue and microvasculature loss in COVID-19 infection

**DOI:** 10.1038/s41598-023-31835-x

**Published:** 2023-03-29

**Authors:** Fritz Gerald P. Kalaw, Alexandra Warter, Melina Cavichini, Darren Knight, Alexandria Li, Daniel Deussen, Carlo Galang, Anna Heinke, Veronica Mendoza, Shyamanga Borooah, Sally L. Baxter, Dirk-Uwe Bartsch, Lingyun Cheng, William R. Freeman

**Affiliations:** 1 Jacobs Retina Center, 9415 Campus Point Drive, La Jolla, CA 92037 USA; 2 Viterbi Family Department of Ophthalmology and Shiley Eye Institute, University of California San Diego, 9415 Campus Point Drive, La Jolla, CA 92037 USA; 3 Department of Ophthalmology, University Hospital, Ludwig-Maximilians-University, 80337 Munich, Germany; 4 Health Department of Biomedical Informatics, University of California San Diego, 9500 Gilman Drive, La Jolla, CA 92093 USA

**Keywords:** Biomarkers, Imaging techniques, Health care

## Abstract

This cross-sectional study aimed to investigate the hypothesis that permanent capillary damage may underlie the long-term COVID-19 sequela by quantifying the retinal vessel integrity. Participants were divided into three subgroups; Normal controls who had not been affected by COVID-19, mild COVID-19 cases who received out-patient care, and severe COVID-19 cases requiring intensive care unit (ICU) admission and respiratory support. Patients with systemic conditions that may affect the retinal vasculature before the diagnosis of COVID-19 infection were excluded. Participants underwent comprehensive ophthalmologic examination and retinal imaging obtained from Spectral-Domain Optical Coherence Tomography (SD-OCT), and vessel density using OCT Angiography. Sixty-one eyes from 31 individuals were studied. Retinal volume was significantly decreased in the outer 3 mm of the macula in the severe COVID-19 group (p = 0.02). Total retinal vessel density was significantly lower in the severe COVID-19 group compared to the normal and mild COVID-19 groups (p = 0.004 and 0.0057, respectively). The intermediate and deep capillary plexuses in the severe COVID-19 group were significantly lower compared to other groups (p < 0.05). Retinal tissue and microvascular loss may be a biomarker of COVID-19 severity. Further monitoring of the retina in COVID-19-recovered patients may help further understand the COVID-19 sequela.

## Introduction

Coronavirus disease 2019 (COVID-19) is an infectious disease caused by severe acute respiratory syndrome coronavirus two (SARS-CoV-2). In December 2019, an outbreak of this respiratory disease emerged in Wuhan, China which was declared a pandemic in March 2020 and has affected many lives since then^1^. As of October 17, 2022, the total number of confirmed cases globally per the World Health Organization (WHO) is 621,797,133, with total deaths of 6,545,561^2^.

The virus affects multiple organ systems aside from the respiratory system^3^. The term “long COVID” was introduced only a few months after the outbreak, as patients reported various symptoms that persisted or developed even after recovery from the infection^4^. Douaud, et al.^5^ investigated brain changes associated with the virus, and one of their notable findings was a greater reduction in the global brain size. A systematic review performed by Castanares-Zapatero, et al.^4^ found that persistent inflammatory processes result in brain microglia activation, as well as pulmonary microvascular damage that may potentially lead to pulmonary hypertension. At the vascular level, the virus promotes an inflammatory state in the vascular endothelium, causing endotheliitis, ultimately leading to a prothrombotic state in the microcirculation^6^. López Reboiro, et al.^7^ suggested that von Willebrand factor (vWF) and lupus anticoagulant (LA), in addition to other molecules, play a crucial role in thrombogenesis induced by this virus. Obtaining serum measurements of vWF and LA may predict permanent endothelial damage, leading to long COVID sequelae such as neuropathic pain or neuropsychiatric issues.

In the eye, several works of literature reported various retinal findings after infection with the novel coronavirus. There is some evidence of vascular pathology associated with COVID-19 infection. The most commonly reported ocular manifestations were retinal hemorrhages and cotton wool spots, while the most common vision-threatening manifestation was retinal vein occlusion with macular edema, making this disease vasculopathic in nature^8^. Several case reports have also diagnosed paracentral acute middle maculopathy, a vasculopathy associated with deep retinal microvascular circulation, after COVID-19 infection using optical coherence tomography (OCT)^9–12^. These are all anecdotal case reports, and many of these patients likely had comorbidities that are not described fully in the papers.

OCT Angiography (OCTA) is a non-invasive imaging technique that provides three-dimensional visualization of the retinal vasculature by moving particles, such as erythrocytes^13^. Although OCTA has been used in assessing retinal vasculature in patients infected with COVID-19^14–27^, some of these studies included patients with systemic confounders, including hypertension and diabetes mellitus, which are known to affect the retinal microvasculature.

The purpose of this study was to investigate the retinal thickness, volume, and vessel density of patients who were diagnosed and recovered from COVID-19 using Spectral Domain OCT (SD-OCT) and OCTA and to compare findings with a control group who had not been infected. We aim to understand the natural course of the ocular findings of this disease. The patients included in this study were carefully selected and had no prior systemic disease that might alter the retinal microvascular architecture.

## Methods

### Design

This cross-sectional, single-institution study was approved by the Institutional Review Board of the University of California San Diego in California, USA, and complied with the Health Insurance Portability and Accountability Act of 1996. All patients provided written informed consent as per institution protocol, and all the data that were collected were anonymized. Data collection and analysis were conducted according to the Principles of the Declaration of Helsinki.

### Participants and patient selection

Participants in the study were subdivided into three groups: (1) Age-matched controls who never developed COVID-19; (2) Patients who received outpatient care after developing mild symptoms (fever and/or cough) and tested positive for COVID-19 by reverse transcription polymerase chain reaction (RT-PCR); (3) Patients who were seen at the University of California San Diego who were diagnosed COVID-19 positive by RT-PCR and required admission to intensive care for further management and respiratory support. Patients in subgroups 2 and 3 were diagnosed with COVID-19 between March 2020 and January 2022 and recovered from the infection, and were clinically stable upon participation in the study. Exclusion criteria were any of the following medical histories before getting infected with COVID-19: primary or secondary hypertension, diabetes mellitus type 1 or 2, hematologic disorders, any types of cancer, any cardiovascular or respiratory disease, ocular history of myopia (> 6 diopters), glaucoma, retinopathies including retinal detachment, retinal vascular disease, macular degeneration, central serous chorioretinopathy, diabetic retinopathy, recent ocular surgery within 6 months of evaluation, and media opacity (corneal, lens, or vitreous) that could affect the image quality of the scans. Participants were initially identified using structured queries of the electronic health record data warehouse (Epic SlicerDicer, Verona, WI, USA), and eligibility was confirmed with each participant via manual chart review and screening interviews regarding the aforementioned inclusion and exclusion criteria prior to study enrollment.

### Examinations and procedures

Participants underwent a comprehensive ophthalmologic examination by trained retina specialists (AL, DK). This included obtaining the best-corrected visual acuity (BCVA) using the Early Treatment Diabetic Retinopathy Study (ETDRS) chart (a standard chart used for measuring visual acuity in ophthalmic research studies, even outside the context of diabetic retinopathy), intraocular pressure (VM), anterior segment examination using slit lamp biomicroscope, and dilated fundus examination using an indirect ophthalmoscope. To assess the macular thickness, volume and vasculature, SD-OCT and OCTA were performed using the Heidelberg HRA + OCT Spectralis System version 1.11.2.0 (Heidelberg Engineering, Heidelberg, Germany). OCT scan pattern were as follows: Number of B-scans = 512, Pattern Size = 3.1 × 3.1 mm/10° × 10°, Distance between B-Scans = 12 μm, ART images average = 7.

### Retinal thickness and volume

**Figure 1:**
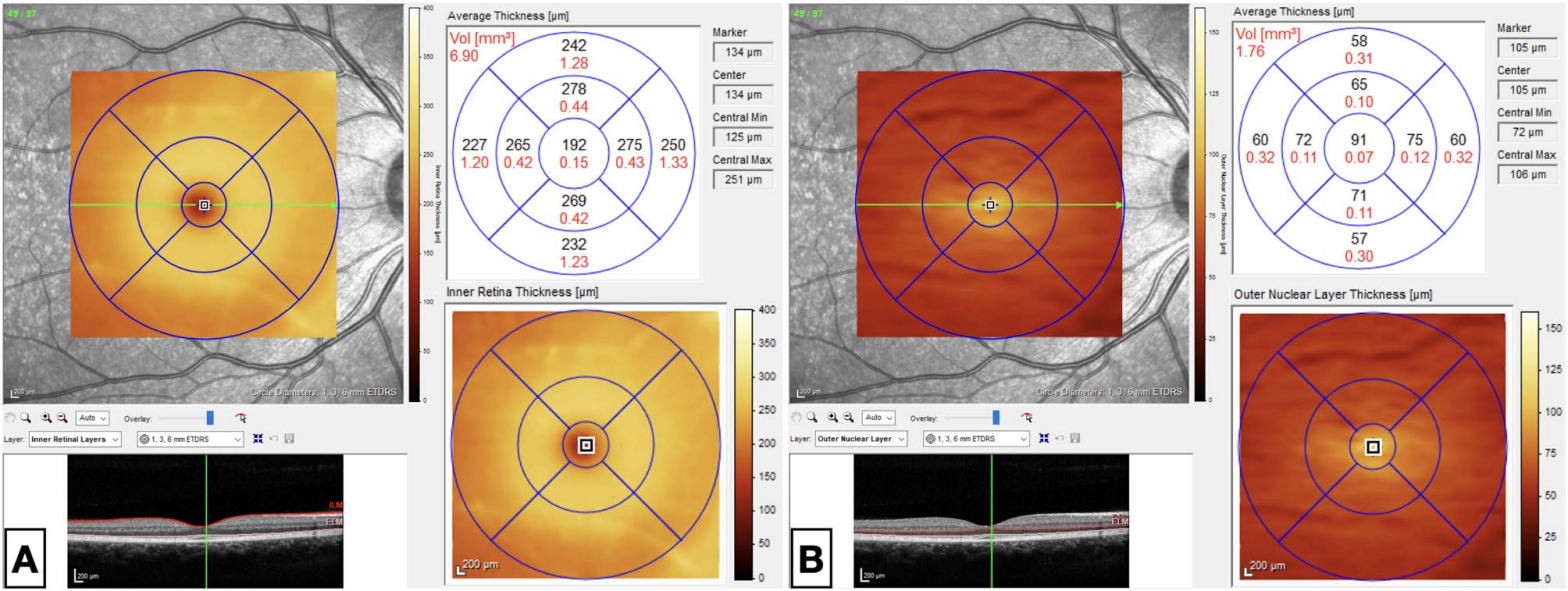
SD-OCT measurement of inner retinal thickness and volume* computed from the difference between (**A**) (Inner retinal layer = ILM + ELM) and (**B**) (Outer nuclear layer = OPL + ELM). *SD-OCT* spectral domain-optical coherence tomography, *ILM* internal limiting membrane, *ELM* external limiting membrane, *OPL* outer plexiform layer. *values of thickness and volume are in μm and mm^3^, respectively.

Retinal thickness and volume were measured using the proprietary Heidelberg HRA + OCT Spectralis software. A thickness map of the central 3 mm of the macula with its corresponding ETDRS sectors was obtained. This map provided nine retinal thickness and volume measurements, which include: central subfield 1 mm, 1 mm inner superior/nasal/inferior/temporal, and 1 mm outer superior/nasal/inferior/temporal. The segmentation was carefully assessed by the investigators, trained retina specialists, before obtaining the numerical data. Inner retinal thickness and volume were computed from the difference of the following thickness map analyses: Inner retinal layer (ILM + ELM) − Outer nuclear layer (OPL + ELM) (Fig. 1).

### Retinal vessel density

Retinal vessel density was analyzed using the AngioTool software version 0.6a (National Institutes of Health, National Cancer Institute, Bethesda, MD, USA) from the acquisition of OCTA^28^. The parameters used were based on a previous study by Choi et al.^29^: threshold parameters = 30 and 255, vessel thickness = 5, removal of small particles = 80. Vessel density was defined as the percentage of area occupied by vessels inside the explant area. Vessel density measurements of the inner retinal layer provided were as follows: Nerve fiber layer vascular plexus (NFLVP), Superficial vascular plexus (SVP), Intermediate capillary plexus (ICP), and Deep capillary plexus (DCP) (Fig. S1^13^).

### Foveal avascular zone

To determine the area, circularity, and roundness of the foveal avascular zone (FAZ), the SVP slab from the OCTA data was analyzed using the proposed method by Shiihara, et al.^30^ using ImageJ version 1.53 k (National Institutes of Health, Bethesda, MD, USA). The FAZ was defined as the region within the SVP slab devoid of hyperreflective signal or flow at the center of the fovea. The border of the zone was manually drawn (FK) (Fig. S2). The parameters included in the FAZ analysis were the following: Area, Perimeter, Fit ellipse, and Feret’s diameter. A circularity value of 1.0 indicates that the FAZ is a perfect circle, and as the value decreases, the less circular or, the more elongated the FAZ is^31^. Roundness refers to the presence or absence of surface irregularities and also refers to the inverse of aspect ratio^32^. Computation for the circularity and roundness was based on the ImageJ manual^31^.

### Statistical analysis

In the current study, retinal vessel density was measured by OCTA from each layer of the inner retina (retina above the outer nuclear layer), including NFLVP, SVP, ICP, and DCP. In addition, inner retinal thickness and retinal volume were also measured from the same area in multiple segments as nine standard ETDRS sectors within the central 10 degrees of the macula. Pooling of vessel density data from NFLVP to the other three layers created a bimodal distribution; therefore, vessel density data from NFLVP was analyzed separately from the other three layers. A bimodal distribution was also present for pooled volume data; therefore, pooled data from the inner five sectors were analyzed separately from the outer four sectors. A generalized linear mixed model was fitted with pooled vessel density (SVP, ICP, and DCP) as responses and location as a random effect nested in eye, patient, and COVID-19 group. Age and sex as covariates were adjusted in the model. A similar mixed model was used to analyze thickness and volume data. All analyses were performed using SAS software 9.4 (SAS Institute, NC, USA).

## Results

A total of 20 patients infected with COVID-19 participated in the study. Thirteen patients developed mild COVID-19 symptoms and received outpatient care, and seven were hospitalized in the intensive care unit, hence considered severe. A control group of 11 healthy patients without COVID-19 was used for comparison and was labeled as normal. The demographics and ocular parameters are summarized in Table 1.

**Table 1:** Demographic characteristics and BCVA across all participants.

	Normal (n = 11)	Mild (n = 13)	Severe (n = 7)
Age (Mean [SD])	38.7 (9.6)	44.6 (6.2)	42 (12.7)
Sex (Female [%])	7 (64%)	8 (62%)	3 (43%)
BCVA in logMAR (Mean [SD])	− 0.085 (0.01)	− 0.048 (0.068)	0.0057 (0.19)

*BCVA* best-corrected visual acuity, *logMAR* logarithm of the minimum angle of resolution*, *SD* standard deviation.

*a logMAR value of 0.1 is equivalent to a 20/25 Snellen chart, 0.0 is equivalent to 20/20, and − 0.1 is equivalent to 20/15.

**Figure 2:**
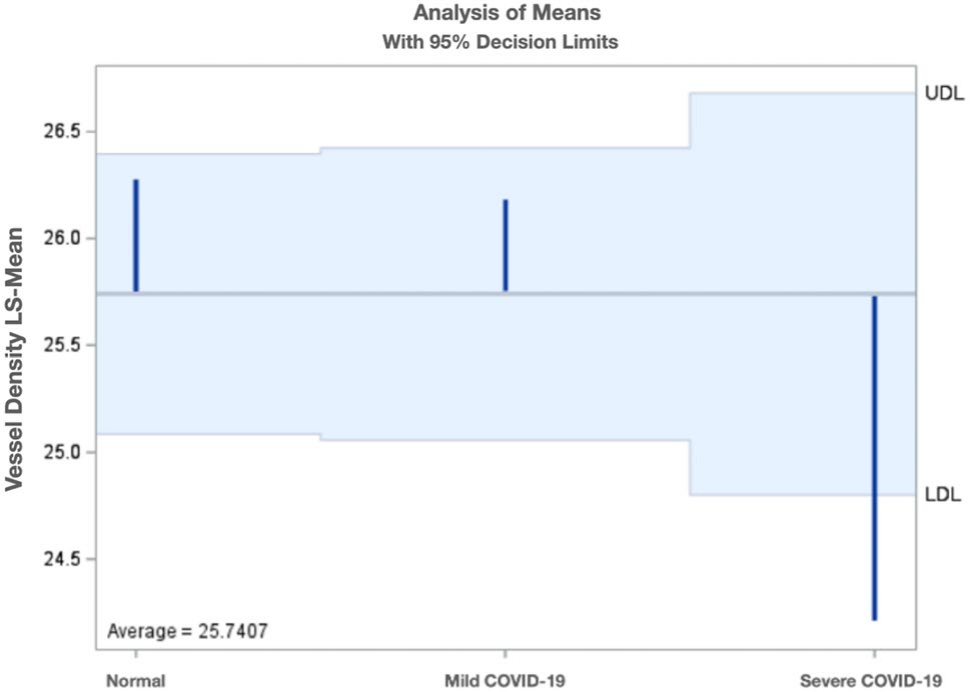
Least square means of the vessel density from the three inner retinal layers stratified by COVID-19 severity.

The analysis revealed that the mean vessel density of pooled data from the three inner retinal layers was significantly lower for severe COVID-19 patients (24.20, least square mean) than that of mild COVID-19 (26.18, p = 0.0057) and normal control patients (26.28, p = 0.004) (Fig. 2). Age and sex were adjusted for, but neither were significant factors. The vessel density of mild COVID-19 patients was not significantly different from that of normal participants (p = 0.87).

**Figure 3:**
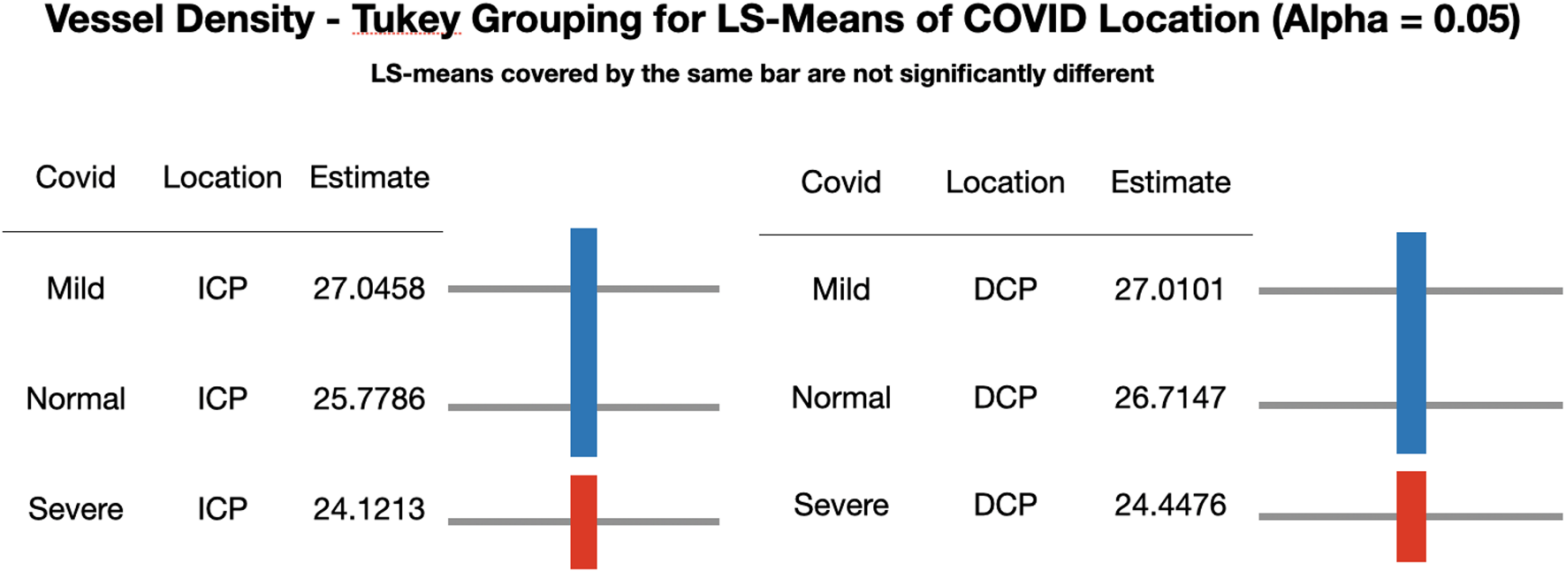
Comparison of least square means of three COVID-19 levels within ICP and DCP. *ICP* intermediate capillary plexus, *DCP* deep capillary plexus, *COVID-19* coronavirus disease-19.

**Figure 4:**
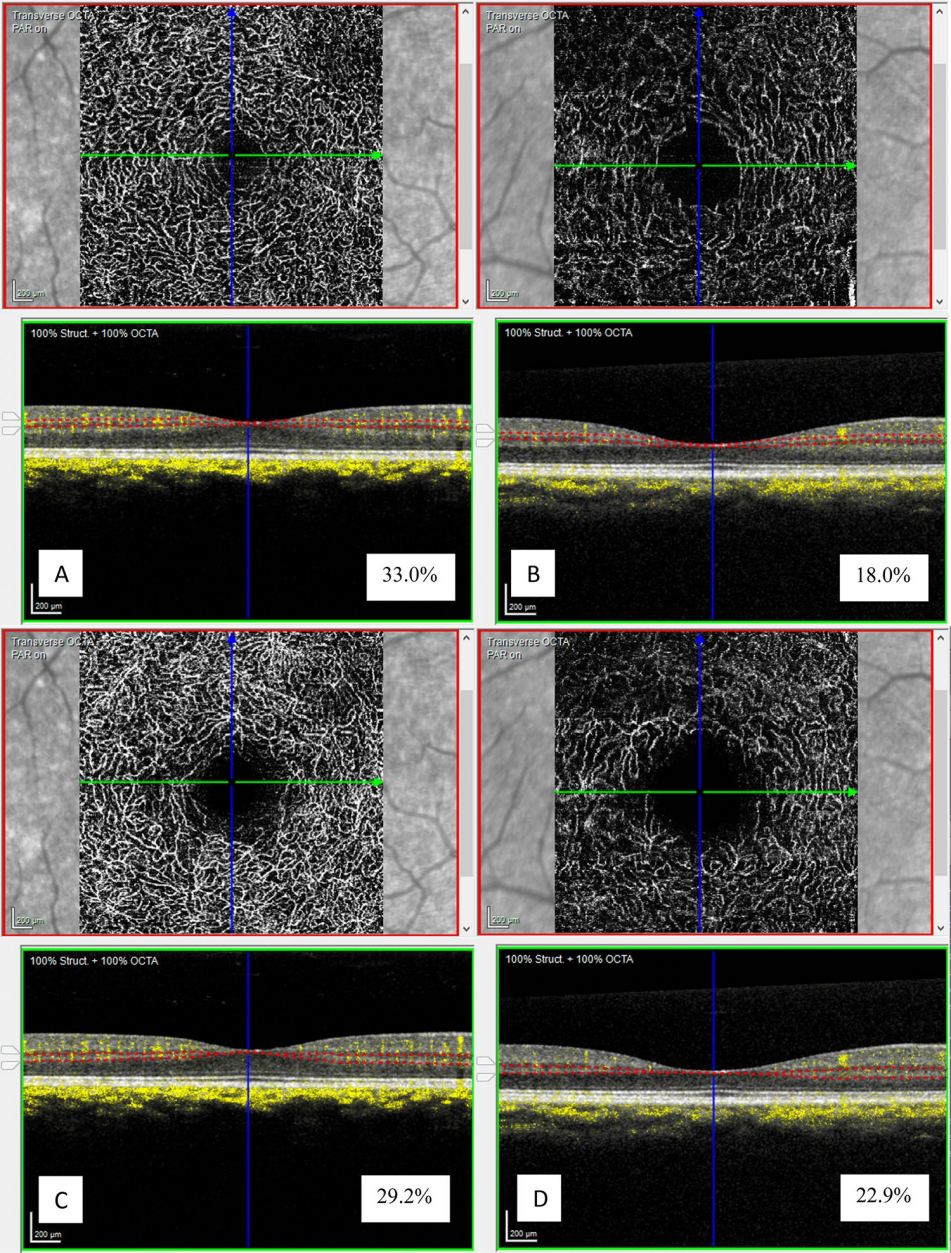
Representative OCTA images of the ICP (**A,B**) and DCP (**C,D**) slabs of normal participant (**A,C**) and severe COVID-19 participant (**B,D**) with their corresponding vessel densities (in percentage). *OCTA* optical coherence tomography angiography, *ICP* intermediate capillary plexus, *DCP* deep capillary plexus, *COVID-19* coronavirus disease-19.

In this generalized linear mixed model, the least square means of the three COVID-19 levels were also compared within each retinal layer. The retinal vessel density of severe COVID-19 patients was significantly lower than that of mild COVID-19 and normal controls for ICP and DCP (p =  < 0.05) (Fig. 3). Figure 4 represents an OCTA ICP and DCP scan of the normal and severe COVID-19 participants. No vessel density difference was seen among the three COVID-19 levels for SVP or NFLVP.

**Figure 5:**
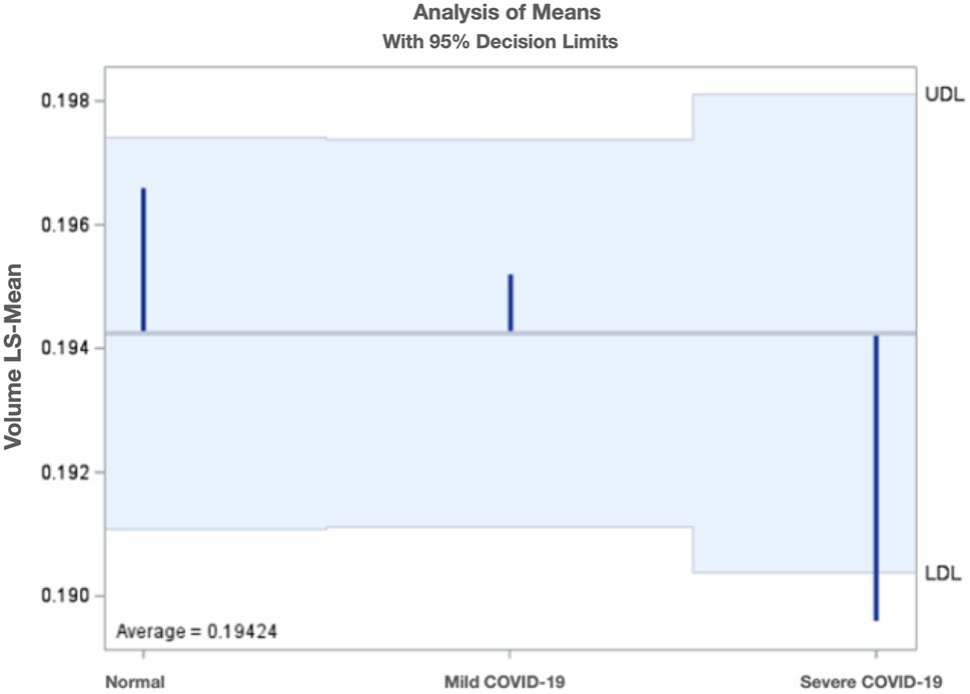
Least square means of the volume from the outer four standard ETDRS sectors stratified by COVID-19 severity. *ETDRS* Early treatment diabetic retinopathy study, *COVID-19* Coronavirus disease-19.

For inner retinal thickness data and volume data, similar analysis did not reveal significant difference between the three COVID-19 groups for the retinal thickness; however, pooled volume from the outer four standard ETDRS sectors revealed a significant lower mean volume for severe COVID-19 patients than the average volume of all patients (p = 0.02) (Fig. 5). The area of foveal avascular zone on OCTA was also analyzed, but no difference was found between the three COVID-19 groups.

In addition, our group performed a sub-analysis to compare mild versus severe COVID-19 patients while adjusting for the interval between the onset of COVID-19 symptoms and days to retinal imaging (SD-OCT and OCTA). The mean interval for mild and severe COVID-19 cases were 151 and 210, respectively. This was not significant and regression analysis revealed that the retinal vascular loss and thinning were not related to the examination interval between symptom onset of COVID-19 and imaging.

## Discussion

Although the SARS-CoV-2 virus primarily affects the respiratory system, the virus has also been noted to exhibit endothelial and neurotropism and thus may infect and damage several organ systems^8^. COVID-19 has been associated with hypoxia and inflammation, which may lead to endothelial dysfunction and coagulation of the small and large vessels in the body^33^. The virus induces chronic oxidative stress in the endothelium, promoting hypercoagulability by releasing vWF. In addition, hypoxic vasoconstriction and direct cellular activation by viral transduction can lead to an increase in blood coagulation parameters^34^. Angiotensin-converting enzyme 2 (ACE2), a metalloproteinase, plays a key role in the SARS-CoV-2 infection as it mediates viral infection and participates in the renin–angiotensin–aldosterone system pathway and development of acute lung injury^35^. In relation to this, neurons and several retinal components, including retinal vascular endothelial cells, Müller cells, ganglion cells, and photoreceptor cells, contain ACE2 receptors. Therefore, retinal tissue, including vessels, may be involved in this infectious process^36^.

There are conflicting results regarding the retinal thickness and FAZ of patients who recovered from this virus. Some groups^37,38^ claimed that the central macular thickness in patients infected with COVID-19 was higher compared to normal controls, whereas other groups^27,36,39^ claimed that there was no difference. Our group compared the inner retinal thickness and volume of the central 3 mm of the macula and found a significant difference in the retinal volume of the outer third of the macula. This is a new finding since there were no available studies to compare retinal volume in COVID-19-recovered patients. Some groups^18,20,23^ also found that the FAZ is larger in patients who recovered from COVID-19 infection compared to normal controls; however, other groups^14,15,21,22,24^ found no difference. Our group found no significant difference between the three groups regarding the FAZ area, circularity, and roundness related to COVID-19 infection or severity.

Prior studies have used OCTA in COVID patients to determine whether retinal vessels are lost or damaged^17,22^. Vessel density has been related to several factors, one of which is age^19^, and the density of the retinal capillary vascular network decreases with older age^40,41^. In addition, co-morbidities such as diabetes and hypertension, which are known to be associated with severe COVID-19 infection, may also affect retinal vessels^42,43^. Our results showed a downward trend of vessel density as age increases, which is consistent among all groups and was corrected for in our analysis. Our results show that severe COVID patients demonstrated an 8% lower overall macular capillary vessel density compared with age-matched controls and those who developed mild COVID-19 symptoms. More particularly, an 8–11% decrease in vessel density was seen in the intermediate and deep capillary plexuses.

Several reports have shown a reduction in vessel density among patients who recovered from COVID-19. These studies noted a decrease in the superficial capillary plexus (SCP)^15–19,21–24,26^ and deep capillary plexus (DCP)^16,17,21–23,26^. Although these results are interesting, certain confounding variables were not accounted for. The severity of the disease or need for hospital admission compared to COVID-19 patients with mild disease was not carefully determined. In addition, some studies also included patients with prior systemic diseases such as hypertension and diabetes mellitus, which may affect the retinal vasculature before developing the COVID-19 infection, and hence, may skew the data. Our study involved careful enrollment of participants with no prior systemic disease (hypertension, diabetes mellitus, cancer, blood disorder), which may affect retinal vascularity and perfusion. Therefore, any effects on retinal vessel density could be more likely attributable to COVID-19 infection. Our group also carefully classified the patients into one of the two categories of recovered COVID-19 patients: those who developed mild symptoms and those who required intensive care admission and oxygen support. Our group found out that the severity of COVID-19 disease is associated with reduced retinal vessel density. Additionally, there was no statistically significant difference in the patient visit and imaging date between mild and severe COVID-19 groups.

To our knowledge, this is the first study that used the Heidelberg Spectralis OCTA to analyze vessel density in carefully characterized patients recovering from COVID-19 and controls. This device subdivides the inner retinal layer into superficial, intermediate, and deep capillary plexus, unlike other OCTA devices, where the superficial and intermediate capillary plexuses are measured as one slab called the superficial plexus. This allows for more detailed phenotypic characterization. Our group found that the intermediate and deep capillary plexuses were significantly affected in COVID-19-infected patients. This may be because the inner layers of the retina have the highest sensitivity to hypoxic stress compared to the outer layers, which are more resistant to hypoxia^44^.

There are several limitations to our study. This study has a relatively small sample size per sub-group since our group excluded patients with systemic co-morbidities like hypertension and diabetes mellitus, which impacted our search for more available participants. This may limit the power to detect small differences between the categories and variables. Compared to retinal thickness and volume measurements, the overall vessel density in the central 3 mm of the macula was analyzed since calculation per ETDRS sector is unavailable. This may dilute regional changes in the macula. In future studies, it would be interesting to analyze OCTA using an ETDRS sector to understand if regional retinal structure correlates with changes in vascularity. Lastly, this is a cross-sectional study and retinal imaging and evaluation were done only once. A longitudinal evaluation of the parameters may be worth exploring in future studies, particularly given recent evidence that COVID-19 can have long-term effects throughout the body.

In conclusion, patients who recovered from COVID-19 after hospitalization showed a significant decrease in retinal tissue, including inner retinal volume and vessel density. Retinal volume and vessel density loss of this magnitude may not cause significant visual symptoms. However, this may be a biomarker for severe COVID or long COVID or COVID damage to the brain and other organs. OCT angiography is a non-invasive technique and can be performed on an undilated pupil. A high-quality exam by a trained operator is estimated to take around five to ten minutes per patient, hence the procedure is undemanding. Close monitoring and surveillance of the patients who recovered from COVID-19 should be considered to assess for long-term sequela of COVID-19 affecting the retina using this non-invasive imaging modality.

## Supplementary Information

Supplementary Figure S1.

Supplementary Figure S2.

## Data Availability

All data in the current study are available from the corresponding author (WRF) upon reasonable request.
