# Supplementary material for: Retinal tissue and microvasculature loss in COVID-19 infection
Source: Sci Rep. 2023 Mar 29;13:5100. doi: 10.1038/s41598-023-31835-x (PMC10050819; doi:10.1038/s41598-023-31835-x)
Supplement: Supplementary file 1 — Supplementary Figure S1. [file 41598_2023_31835_MOESM1_ESM.pdf]

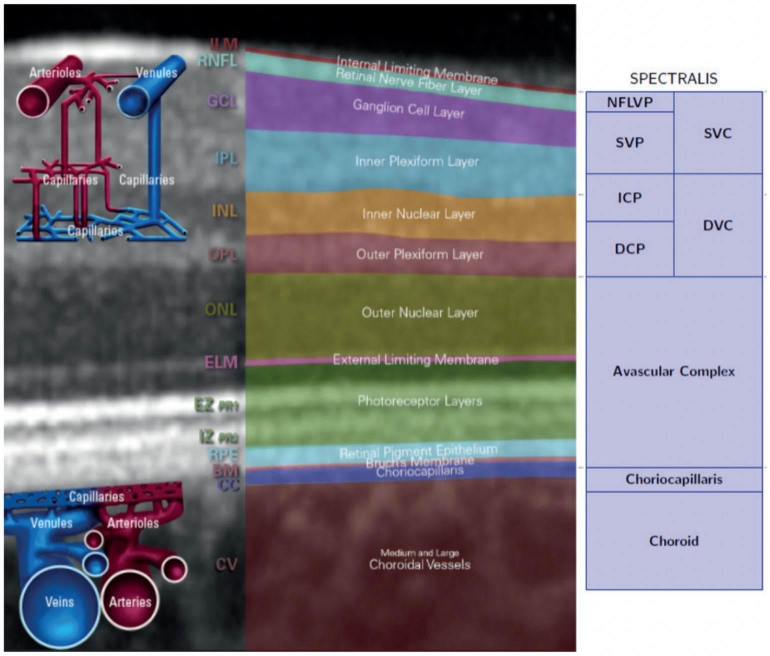

Figure S1. Heidelberg Spectralis SD-OCT and OCTA layers.
SD-OCT – Spectral-Domain Optical coherence tomography, NFLVP – Nerve fiber layer vascular plexus, SVP – Superficial vascular plexus, ICP – Intermediate capillary plexus, DCP – Deep capillary plexus, SVC – Superficial vascular complex, DVC – Deep vascular complex
*Figure was obtained with permission from Heidelberg Engineering - Heidelberg Spectralis OCTA manual[35]
