# Supplementary material for: Retinal tissue and microvasculature loss in COVID-19 infection
Source: Sci Rep. 2023 Mar 29;13:5100. doi: 10.1038/s41598-023-31835-x (PMC10050819; doi:10.1038/s41598-023-31835-x)
Supplement: Supplementary file 2 — Supplementary Figure S2. [file 41598_2023_31835_MOESM2_ESM.pdf]

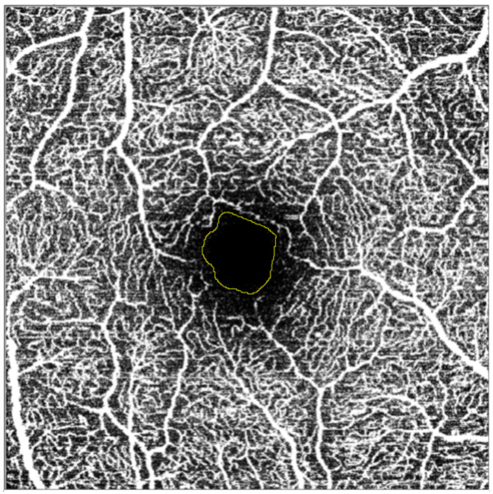

Figure S2. Manual measurement of the FAZ using the ImageJ software. The image was obtained from the SVP slab of the OCTA. The encircled yellow area corresponds to the region devoid of flow or hyperreflective signal.

FAZ – Foveal avascular zone, SVP – Superficial vascular plexus, OCTA – Optical coherence tomography angiography
